# The educational impact of Mini-Clinical Evaluation Exercise (Mini-CEX) and Direct Observation of Procedural Skills (DOPS) and its association with implementation: A systematic review and meta-analysis

**DOI:** 10.1371/journal.pone.0198009

**Published:** 2018-06-04

**Authors:** Andrea C. Lörwald, Felicitas-Maria Lahner, Zineb M. Nouns, Christoph Berendonk, John Norcini, Robert Greif, Sören Huwendiek

**Affiliations:** 1 Department of Assessment and Evaluation, Institute of Medical Education, University of Bern, Bern, Switzerland; 2 FAIMER, Philadelphia, Pennsylvania, United States of America; 3 Department of Anaesthesiology and Pain Therapy, Bern University Hospital, University of Bern, Bern, Switzerland; Stanford University School of Medicine, UNITED STATES

## Abstract

**Introduction:**

Mini Clinical Evaluation Exercise (Mini-CEX) and Direct Observation of Procedural Skills (DOPS) are used as formative assessments worldwide. Since an up-to-date comprehensive synthesis of the educational impact of Mini-CEX and DOPS is lacking, we performed a systematic review. Moreover, as the educational impact might be influenced by characteristics of the setting in which Mini-CEX and DOPS take place or their implementation status, we additionally investigated these potential influences.

**Methods:**

We searched Scopus, Web of Science, and Ovid, including All Ovid Journals, Embase, ERIC, Ovid MEDLINE(R), and PsycINFO, for original research articles investigating the educational impact of Mini-CEX and DOPS on undergraduate and postgraduate trainees from all health professions, published in English or German from 1995 to 2016. Educational impact was operationalized and classified using Barr’s adaptation of Kirkpatrick’s four-level model. Where applicable, outcomes were pooled in meta-analyses, separately for Mini-CEX and DOPS. To examine potential influences, we used Fisher’s exact test for count data.

**Results:**

We identified 26 articles demonstrating heterogeneous effects of Mini-CEX and DOPS on learners’ reactions (Kirkpatrick Level 1) and positive effects of Mini-CEX and DOPS on trainees’ performance (Kirkpatrick Level 2b; Mini-CEX: *standardized mean difference (SMD)* = 0.26, *p* = 0.014; DOPS: *SMD* = 3.33, *p*<0.001). No studies were found on higher Kirkpatrick levels. Regarding potential influences, we found two implementation characteristics, “quality” and “participant responsiveness”, to be associated with the educational impact.

**Conclusions:**

Despite the limited evidence, the meta-analyses demonstrated positive effects of Mini-CEX and DOPS on trainee performance. Additionally, we revealed implementation characteristics to be associated with the educational impact. Hence, we assume that considering implementation characteristics could increase the educational impact of Mini-CEX and DOPS.

## Introduction

Since the introduction of the Mini Clinical Evaluation Exercise (Mini-CEX) in 1995 and the Direct Observation of Procedural Skills (DOPS) in 2003, their use has spread rapidly around the world [1–3]. Mini-CEX and DOPS are two commonly used workplace-based assessments [4], which consist of a direct observation and a feedback conversation [5]. Workplace-based assessments are assessments of trainees’ performance in the workplace. Thus, in contrast to many other assessments in medical education, these assessments do not occur in artificial settings, but take place as part of the daily work. Another feature of workplace-based assessments is that they offer the opportunity to provide trainees with feedback on their performance. Therefore, they play an important role in competency-based medical education [4, 6]. In the Mini-CEX, the trainee is evaluated regarding history taking, physical examination skills, communication skills, clinical judgment, professionalism, organization/efficiency, and overall clinical care [5]. In the DOPS, the focus lies on procedural skills. Here, the trainee is evaluated regarding his or her demonstrated understanding of indications, relevant anatomy, technique of procedure, obtaining informed consent, demonstrating appropriate preparation pre-procedure, technical ability, aseptic technique, seeking help where appropriate, post-procedure management, communication skills, consideration of patient/professionalism, and overall ability to perform the procedure [5].

Besides medical training, Mini-CEX and DOPS have also been introduced in other health professions, such as nursing, midwifery or dentistry [7–9]. The opportunity for direct observation of trainees in their clinical workplace makes Mini-CEX and DOPS authentic measures of performance and clinical competence. Direct observation of trainees’ performance provides a convenient opportunity to give feedback on observed behavior [10, 11]. Thus, the concept of Mini-CEX and DOPS appears reasonable for assessing clinical competence and for individually supporting trainees’ development through the provision of feedback [5].

Due to their feedback components, Mini-CEX and DOPS are increasingly used as methods of formative assessment, with the goal of shaping and supporting trainees’ learning [5, 12]. Data on the consequences of Mini-CEX and DOPS, namely support of trainees’ learning, would constitute an important source of their validity [13]. In order to demonstrate such consequential validity, it is necessary to investigate the impact of the assessment, namely whether it is beneficial or harmful, intended or unintended [13]. With a main focus on the intended effects, Miller and Archer [14] performed a systematic review on the educational impact of Mini-CEX, DOPS, case based discussion, and multisource feedback, analyzing whether these workplace-based assessments actually support trainees’ learning. Educational impact was operationalized by applying Barr’s adaptation of Kirkpatrick’s four-level model [15]. This model is commonly used in medical education in order to evaluate educational programs, and was also used in other studies evaluating the educational impact of Mini-CEX/DOPS [14]. By applying Barr’s adaptation of Kirkpatrick’s four-level model, the educational impact can be analyzed on different levels: Level 1 describes learners’ reactions, Level 2a includes modification of attitudes/perceptions, Level 2b addresses the acquisition of knowledge/skills, Level 3 analyzes change in behavior, Level 4a is concerned with change in organizational practice, and Level 4b comprises benefits to patients/clients [15]. Whereas for multisource feedback, Miller and Archer [14] found evidence demonstrating positive effects on trainees’ performance (Level 2b), this was not the case for Mini-CEX and DOPS. With regard to Mini-CEX and DOPS, all articles investigated postgraduate medical trainees’ perceptions of the tools (Level 1) rather than outcomes such as change in behavior or benefits for patients. As the available evidence on the educational impact of Mini-CEX and DOPS was scarce in 2010, and was limited to postgraduate medical trainees and outcomes on Kirkpatrick Level 1 [14], it would be interesting to ascertain whether more evidence has been gathered in the interim, especially on higher Kirkpatrick levels, including evidence on undergraduate medical trainees and trainees in other health professions.

Mini-CEX and DOPS should be used in the workplace during daily clinical work. However, it has been shown that the assessments are perceived as additional workload [16]. Hence, implementation of Mini-CEX and DOPS is challenging, which could affect their educational impact. In another context, Durlak and DuPre [17] demonstrated that the extent to which a program is implemented successfully significantly influences the outcomes of that program. Within implementation, Durlak and DuPre [17] distinguish eight characteristics: 1) fidelity (the extent to which the innovation corresponds to the originally intended program), 2) dosage (how much of the original program has been delivered), 3) quality (how well different program components have been conducted), 4) participant responsiveness (the degree to which the program stimulates the interest or holds the attention of participants), 5) program differentiation or program uniqueness (the extent to which a program’s theory and practices can be distinguished from other programs), 6) monitoring of control (which involves describing the nature and amount of services received by members of the control groups), 7) program reach (the rate of involvement and representativeness of program participants) and 8) adaptation (which refers to changes made in the original program during implementation). As all these characteristics might be relevant for the educational impact of Mini-CEX and DOPS, it would be interesting to consider characteristics of the setting and implementation status as potential influences.

Thus, we aim to answer the following research questions:

1. “What evidence is there that Mini-CEX and DOPS have an educational impact on undergraduate and postgraduate trainees in the health professions?”
2. “Are characteristics of the setting in which Mini-CEX and DOPS take place, or the implementation status of Mini-CEX/DOPS associated with the educational impact of the tools?”

Demonstrating the educational impact of Mini-CEX and DOPS in a systematic review would be helpful for educators in terms of justifying the use of the tools in health professions training. Knowing which characteristics of the setting and of the implementation status influence the educational impact of Mini-CEX and DOPS would advance our understanding of when and how to use Mini-CEX and DOPS in order to increase their educational benefits.

## Methods

To guide our work, we used the recommendations of Cook and West [18] and followed their seven key steps, from defining a focused question to analyzing and synthesizing the results.

For the first step, we defined the focused research questions as stated at the end of the introduction using the PICO statement. As population, we defined undergraduate and postgraduate trainees in the health professions; as intervention, we defined Mini-CEX and DOPS; as outcome, we defined educational impact operationalized using Barr’s adaptation of Kirkpatrick’s four-level model. We did not define any comparison. To answer the first research question and to summarize all of the existing literature, we chose a systematic review as the most suitable strategy (step II). To answer our second research question, we extracted the relevant data from the studies included and analyzed whether there are associations of the educational impact with characteristics of the setting and with characteristics of the implementation status. The study protocol (http://dx.doi.org/10.17504/protocols.io.nmpdc5n) and data (https://doi.org/10.6084/m9.figshare.6275288.v1) are available online.The authors did not receive any specific funding for this review. A PRISMA checklist was completed and adhered to (S1 Table).

### Literature search (data sources and search terms)

In the third step, we assembled a team including a librarian. In order to identify relevant articles as comprehensively as possible, the search strategy was developed and discussed in close collaboration with our librarian. Together, we decided to use the following electronic databases: Scopus, Web of Science (all databases), and Ovid, including All Ovid Journals, Embase, ERIC, Ovid MEDLINE(R), and PsycINFO (step IV). Search terms were "mini-CEX" OR "mini clinical evaluation exercise" OR "direct observation of procedural skills" OR "work-based assessment" OR "workplace-based assessment" OR “supervised learning event” OR “supervised learning events”. We analyzed all articles published between the first description of Mini-CEX in 1995/DOPS in 2003 and December 2016 (date of access: 02.12.2016). Hits from the different databases were exported to an EndNote library and duplicates were removed.

### Article selection

To identify articles relevant to our research question, we defined the following selection criteria (step V):

1. Article type: Original research articles were included; other article types such as reviews, conference abstracts, letters or editorials were excluded.
2. Language: Articles in English and German were included; articles in other languages were excluded.
3. Population: Undergraduate and postgraduate trainees from all health professions were included.
4. Object of investigation/intervention: Articles investigating Mini-CEX or DOPS provided by experts in real-life clinical encounters were included; other interventions such as Multisource Feedback, case based discussions or Mini-CEX and DOPS used in simulation settings were excluded.
5. Outcome: Articles describing the educational impact of Mini-CEX or DOPS, which was operationalized using Barr’s adaptation of Kirkpatrick’s four-level model [15], were included.

Two authors independently scanned the articles for eligibility. Differences in article selection were discussed until consensus was reached. Articles were selected in two rounds: First, titles and abstracts were scanned, and second, remaining articles were selected based on full texts. In the first round, 63 out of 1478 articles were not in consensus and had to be discussed based on the full text. Based on the full text, inclusion/exclusion was clear and all articles were classified unanimously. To visualize the review process, we used the four-phase flow diagram as recommended in the PRISMA statement [19].

### Data extraction

From the articles included in our review, the authors extracted the following key information (step VI): setting (institution, Mini-CEX/DOPS, purpose of Mini-CEX/DOPS, feedback recipient, mandatory Mini-CEX/DOPS, number of Mini-CEX/DOPS, assessment sheet, feedback provider, similar tools), study design (aim of study, method, intervention, control, sample), implementation status (fidelity, dosage, quality, participant responsiveness, program differentiation, monitoring of control, program reach, adaptation) [17], and reported outcome.

### Data analysis

As a final step, the authors analyzed the data regarding the quality of the studies, educational impact, and the potential influences on the educational impact (step VII). Effects of Mini-CEX and DOPS were analyzed separately. Articles describing the educational impact of both Mini-CEX and DOPS were separated into individual studies.

#### Study quality

The methodological quality of the studies was assessed using the MERSQI (Medical Education Research Study Quality Instrument) [20]. The MERSQI consists of ten items and takes into account study design (single-group cross-sectional or single-group posttest only: 1 point; single-group pretest and posttest: 1.5 points; nonrandomized, two groups: 2 points; randomized controlled trial: 3 points), sampling (Institutions: one institution: 0.5 points; two institutions: 1 point; three or more institutions: 1.5 points. Response rate: < 50% or not reported: 0.5 points; 50%–74%: 1 point; ≥ 75%: 1.5 points), type of data (assessment by study participant: 1 point; objective: 3 points), validity evidence for evaluation instrument scores (content validity: 1 point; validity evidence on internal structure: 1 point; relationships to other variables: 1 point), data analysis (Sophistication: descriptive analysis only: 1 point; beyond descriptive analysis: 2 points. Appropriateness: data analysis appropriate for study design and type of data: 1 point), and outcomes (satisfaction, attitudes, perceptions, opinions, general facts: 1 point; knowledge, skills: 1.5 points; behaviors: 2 points; patient/health care outcome: 3 points). The MERSQI demonstrated high interrater reliability (0.72–0.98) and a strong association with the 3-year citation rate and journal impact factors [20]. The minimum MERSQI score is 5 and the maximum is 18.

#### Educational impact

Reported outcomes were classified according to Barr’s adaptation of Kirkpatrick’s four-level model [15]. The model consists of level 1 (learner’s reaction), level 2a (modification of attitudes/perceptions), level 2b (acquisition of knowledge/skills), level 3 (change in behavior), level 4a (change in organizational practice), and level 4b (benefits to patients/clients) [15]. We used this framework in a strict manner and classified self-assessed improvements in performance into Kirkpatrick level 1 (learner’s reaction).

For meta-analysis, effect sizes were calculated using Hedges’ g (standardized mean differences) for each comparison [21]. If information reported in the articles was insufficient, we requested means and standard deviations from the corresponding authors via e-mail or ResearchGate. To quantify heterogeneity across studies, we used the *I*^*2*^- and *tau*^*2*^- statistics [22]. A random effects model was used to pool weighted effect sizes.

#### Potential influences

As potential influences on the educational impact of Mini-CEX/DOPS, we considered characteristics of the setting and of the implementation status [17]. Within the setting, we considered the purpose for which Mini-CEX/DOPS was used, whether the use of Mini-CEX/DOPS was mandatory, and what the assessment sheet looked like. Within the implementation status, we considered fidelity, dosage, quality, participant responsiveness, program differentiation, monitoring of control, program reach, and adaptation. To perform the analyses, implementation characteristics were translated to the context of Mini-CEX and DOPS and manifestations were defined (high, medium, low; or yes or no). Since there were no pre-existing definitions of manifestations of implementation characteristics, we had to define these ourselves. Thus, the definitions were partly influenced by the data reported in the studies, e.g. to decide on the level of program differentiation (the extent to which Mini-CEX/DOPS can be distinguished from other programs such as in-training evaluation reports, case based discussion, or multisource feedback), we first examined which other programs were described in the articles. Based on this information, we discussed their similarity with Mini-CEX/DOPS and defined three manifestations of program differentiation, as described in S2 Table. We discussed this operationalization until the whole research team agreed on its appropriateness. According to these categories, two authors classified the single characteristics’ manifestations for each study.

To analyze whether there is an association of the educational impact with characteristics of the setting and with characteristics of the implementation status of Mini-CEX/DOPS, we used Fisher’s exact test for count data [23]. For statistical analyses, we used R [24] and the R package “meta” [25]. Statistical significance was defined by a 2-sided *α* < 0.05.

We assume a negligible risk of bias across and within studies. One major bias across studies is the publication bias favoring the publication of studies demonstrating positive results. With regard to Mini-CEX and DOPS, however, a relevant proportion of studies show negative results [26–29]. We thus assume negligible publication bias in this review. With regard to the study design, there might be bias within studies. For example, some studies were sequential cohort studies, with limited randomization. In the meta-analyses, however only studies with compatible study designs and compatible bias within the studies were pooled. Thus, we did not further adjust for potential bias within studies.

## Results

### Search results

**Fig 1:**
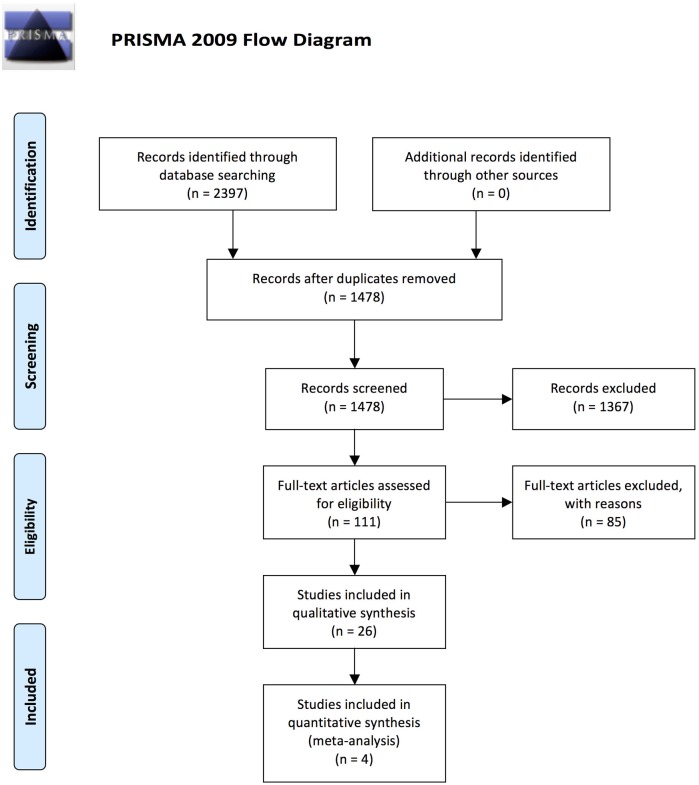
Flow diagram of search results according to the PRISMA statement. *From*: Moher D, Liberati A, Tetzlaff J, Altman DG, The PRISMA Group (2009). *P*referred *R*eporting *I*terns for *S*ystematic Reviews and *M*eta-*A*nalyses: The PRISMA Statement. PLoS Med 6(7): e1000097. doi:10.1371/journal.pmed1000097. **For more information, visit** www.prisma-statement.org.

Our search identified 2397 records. After duplicates were removed, 1478 records remained. By screening titles and abstracts, 1367 records were removed. The remaining 111 records were assessed based on their full text. Of these, 85 articles were excluded, and 26 articles met all eligibility criteria and were included in our review (Fig 1).

Seventeen articles were on Mini-CEX [7, 8, 26, 27, 30–42], six were on DOPS [9, 29, 43–46], and three investigated both Mini-CEX and DOPS [28, 47, 48]. In the following, study outcomes are described according to Kirkpatrick levels. Extracted data of all articles are presented in S3 Table.

### Outcomes according to Kirkpatrick levels

#### Outcomes on Kirkpatrick level 1 (learner’s reaction)

Eighteen articles investigated the effect of Mini-CEX on Kirkpatrick level 1 [7, 8, 26–28, 30–32, 34–37, 39–42, 47, 48]. These studies aimed to evaluate the implementation of Mini-CEX and to assess user experiences and satisfaction with Mini-CEX. As shown in Table 1, eleven studies reported high educational impact (high satisfaction with Mini-CEX; trainees perceived Mini-CEX as helpful or very helpful for learning), four studies reported medium educational impact (moderate satisfaction with Mini-CEX; Mini-CEX was perceived as rather helpful or rather not helpful for medical training), and three studies reported low educational impact (low satisfaction with Mini-CEX; Mini-CEX was not perceived as helpful for medical training). For detailed information on the studies, please refer to S3 Table.

**Table 1:** Numbers of studies with low, medium, and high educational impact on Kirkpatrick level 1, separately for Mini-CEX and DOPS.

	Educational impact on Kirkpatrick level 1		
	low	medium	high
Mini-CEX	3 (17%) [26–28]	4 (22%) [31, 36, 42, 47]	11 (61%) [7, 8, 30, 32, 34, 35, 37, 39–41, 48]
DOPS	2 (28.5%) [28, 29]	3 (43%) [43, 46, 47]	2 (28.5%) [45, 48]

Seven studies reported effects of DOPS on Kirkpatrick level 1 [28, 29, 43, 45–48]. Aims of the studies were to evaluate the implementation of DOPS, user experiences and their satisfaction with the tool.

Two studies reported high educational impact (high satisfaction with DOPS; DOPS was perceived as useful), three studies reported medium educational impact (moderate satisfaction with DOPS; DOPS was perceived as rather useful or not useful), and two studies reported low educational impact (DOPS was not perceived as useful). For detailed information on the studies, please refer to S3 Table.

#### Outcomes on Kirkpatrick level 2a (modification of attitudes and perceptions)

We found no studies reporting an educational impact of Mini-CEX or DOPS on Kirkpatrick level 2a.

#### Outcomes on Kirkpatrick level 2b (acquisition of knowledge and skills)

Two articles, encompassing three studies, reported effects of Mini-CEX on Kirkpatrick level 2b [33, 38]. These studies aimed to investigate the effect of Mini-CEX on trainees’ performance. Study designs were sequential cohort studies. The study of Kim, Willett [33] compared mandatory formative Mini-CEX to no or voluntary Mini-CEX, measuring undergraduate medical trainees’ performance in a summative end-of-year objective structured clinical examination. The other two studies by Suhoyo, Schönrock-Adema [38] compared mandatory formative and summative Mini-CEX to the existing assessment program and measured undergraduate medical trainees’ performance in an objective structured long examination record.

**Fig 2:**
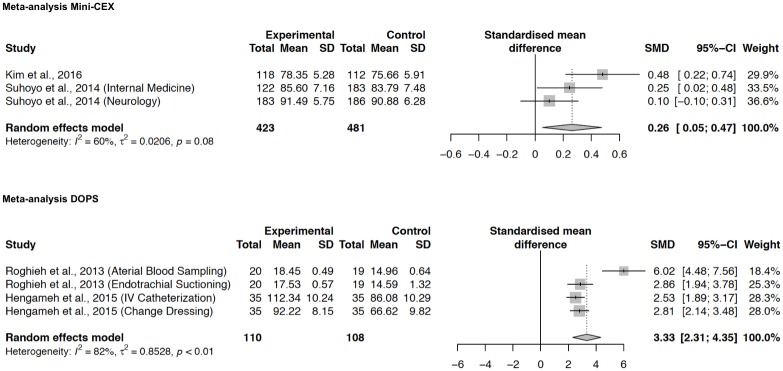
Random-effects meta-analyses on the effects of Mini-CEX or DOPS compared with no intervention on trainee performance. Positive numbers favor Mini-CEX/DOPS. Abbreviation used: *SMD (standardized mean difference)*.

Educational impact was high in two studies and medium in one study. Effect sizes ranged from 0.10 to 0.48 and were pooled in a meta-analysis, which revealed a significant *standardized mean difference (SMD)* of 0.26 (95% *CI*, 0.05–0.47; *p* = 0.014) (Fig 2). Effect sizes of the single studies were not heterogeneous (*I*^*2*^ = 60%, *tau*^*2*^ = 0.0206, *p* = 0.08).

Two articles reported on the educational impact of DOPS on Kirkpatrick level 2b [9, 44]. These studies were both randomized controlled trials. Hengameh, Afsaneh [44] evaluated the effect of formative DOPS on undergraduate nursing trainees’ performance and compared it to the routine method, which included a subjective judgment of trainees’ general skills. As outcome measures, they used validated and reliable assessment checklists for two procedures: intravenous catheterization and changing dressing. Roghieh, Fateme [9] investigated the effect of mandatory formative DOPS on undergraduate nursing trainees’ performance. In their study, DOPS was performed in addition to the routine assessment program to which it was compared. Outcomes were measured using two checklists, one for trainees’ skill levels in arterial blood sampling and one for trainees’ skill levels in endotracheal suctioning.

Educational impact was high in both studies. As both studies reported two outcome measurements each, two effect sizes were calculated for each study. Effect sizes ranged from 2.53 to 6.02. Pooling effect sizes in a meta-analysis revealed an *SMD* of 3.33 (95% *CI*, 2.31–4.35; *p*<0.001) (Fig 2). Effect sizes of the single studies were significantly heterogeneous (*I*^*2*^ = 82%, *tau*^*2*^ = 0.8528, *p*<0.01).

#### Outcomes on Kirkpatrick level 3 (change in behavior), 4a (change in organizational practice) and 4b (benefits to clients or patients)

We found no studies reporting effects of Mini-CEX or DOPS on Kirkpatrick levels 3 or 4.

### Analysis of potential influences on the educational impact

#### Mini-CEX

Our analysis revealed that the educational impact of Mini-CEX was positively associated with two characteristics of the implementation status: quality of Mini-CEX (*n* = 9, *p* = 0.004) and participant responsiveness (*n* = 17, *p*<0.001) (Tables 2 and 3). No significant links to the educational impact were found with regard to the purpose of Mini-CEX (*n* = 18, *p* = 0.666), its mandatory use (*n* = 20, *p* = 0.569), the assessment sheet (*n* = 16, *p* = 1.000), fidelity (*n* = 2, *p* = 1), dosage I (*n* = 9, *p* = 1.000), dosage II (*n* = 9, *p* = 1.000), program differentiation (*n* = 13, *p* = 0.351), monitoring of control (*n* = 3, *p* = 1.000), program reach (*n* = 12, insufficient data to calculate *p*), and adaptation (*n* = 1, insufficient data to calculate *p*).

**Table 2:** Cross table visualizing the association between quality and educational impact.

		Quality			
		high	medium	low	Sum
Educational impact	high	4	0	0	4
	medium	1	1	0	2
	low	0	0	3	3
	Sum	5	1	3	9

**Table 3:** Cross table visualizing the association between participant responsiveness and educational impact.

		Participant responsiveness			
		high	medium	low	Sum
Educational impact	high	9	1	0	10
	medium	0	3	1	4
	low	0	0	3	3
	Sum	9	4	4	17

#### DOPS

Analyzing the studies that investigated DOPS, no significant links between the setting or the implementation status and the educational impact were found. There was no significant link between the educational impact and the mandatory use of DOPS (*n* = 6, *p* = 1), participant responsiveness (*n* = 6, *p* = 0.2), or program differentiation (*n* = 7, *p* = 0.114). With regard to the purpose of DOPS, the assessment sheet, fidelity, dosage I and II, quality, monitoring of control, program reach, and adaptation, the reported data were insufficient to calculate Fisher’s exact test for count data.

## Discussion

In this systematic review, we summarized the available evidence on the educational impact of Mini-CEX and DOPS from 1995 to 2016 and analyzed associations of the educational impact with characteristics of the setting and characteristics of the implementation status. We identified 26 articles, 17 on Mini-CEX, six on DOPS, and three on both tools. The majority of the articles investigated effects of Mini-CEX/DOPS on learners’ reactions (Kirkpatrick level 1), showing mixed results. Four articles reported effects on trainee performance (Kirkpatrick level 2b) and were synthesized into two meta-analyses: one on DOPS and one on Mini-CEX. Both meta-analyses revealed a positive effect of Mini-CEX or DOPS on trainee performance. We found two characteristics of implementation status to be associated with the educational impact: quality and participants’ responsiveness.

In the meta-analyses the effect of DOPS was about ten times higher than that of Mini-CEX [9, 33, 38, 44]. This might be due to the different study designs: Whereas the studies on DOPS focused on specific procedures and aligned intervention and outcome measurement, the studies on Mini-CEX used a more general approach and did not match intervention and outcome measurement as closely. In the studies on DOPS, trainees received feedback on their performance in predefined procedures, and afterwards, performance in exactly these procedures was assessed and compared to the control. In the studies on Mini-CEX, the tool could be used for any situation within trainees’ internships, and the outcome measurements were more general, using the regular end-of-term assessments. Hence, in the studies on Mini-CEX, a large proportion of the effect could have remained invisible.

Since the educational impact in the meta-analysis on DOPS as well as the reported outcomes on Kirkpatrick level 1 on Mini-CEX and DOPS varied, we examined whether the setting in which the tools take place and their implementation status affect their educational impact. In fact, we found two associations between the implementation status of Mini-CEX and its educational impact: quality and participant responsiveness. The correlation between the quality of implementation and the educational impact indicates that low quality is associated with low educational impact and high quality is associated with high educational impact. In other words, the proper conducting of Mini-CEX, including a realistic reflection of trainee performance during direct observation, followed by a constructive feedback conversation, seems to be a prerequisite for its educational impact [49–51]. This might sound logical, but indicates the importance of implementation [17, 52]. The positive association between participant responsiveness and the educational impact of Mini-CEX most likely stems from their strongly overlapping definitions. Since no study on Kirkpatrick level 2b commented on participant responsiveness, only studies on Kirkpatrick level 1 were entered into the analysis. Thus, the definitions of the educational impact on Kirkpatrick level 1 and participant responsiveness are very similar. Both are concerned with satisfaction and perceived helpfulness of the tools. The only difference is that the educational impact focuses on trainees’ perspectives, and participant responsiveness considers the perspectives both of trainees and supervisors. Therefore, the question of whether the positive association between participant responsiveness and educational impact also holds true for higher Kirkpatrick levels remains open and should be considered in future studies. Besides the two positive associations with participant responsiveness and quality, no other characteristics of the setting and the implementation status showed significant associations with the educational impact of Mini-CEX. With regard to DOPS, no significant associations were found at all. This might be due to the limited data base, as on average, only three out of eight characteristics of the implementation status were reported.

As hypothesized, our study demonstrates that proper implementation is positively associated with the educational impact of Mini-CEX. This finding has several implications for practitioners and researchers in health professions education. For practitioners, our results suggest that it is essential to assure proper implementation and integration of the assessments into daily routine in order for them to have an educational impact. For researchers, it is crucial to take implementation characteristics into account when evaluating the educational impact of Mini-CEX and DOPS. For example, a negative result, such as no effect of Mini-CEX/DOPS on trainees’ performance, might be explained by improper implementation of the tools. Addressing implementation characteristics, such as fidelity, dosage, quality, participant responsiveness, program differentiation, monitoring of control, program reach, and adaptation, when evaluating the tools can also help in improving the educational impact in the future. To facilitate further research on factors influencing the educational impact, rigorous reporting is necessary. Above and beyond the usual reporting standards [53, 54], the implementation framework of Durlak and DuPre [17], as applied in this systematic review, could be useful to improve the reporting of educational interventions and to guide implementation processes. Since the available evidence was limited to Kirkpatrick levels 1 and 2b, further research should also consider effects of Mini-CEX and DOPS on higher Kirkpatrick levels, such as changes in behavior (Kirkpatrick Level 3), organizational practice (Kirkpatrick Level 4a), or benefits to patients (Kirkpatrick Level 4b). The evidence on Kirkpatrick level 2b was limited to undergraduate trainees. Therefore, it would be interesting to examine whether the positive effects of Mini-CEX and DOPS also hold true regarding postgraduate trainees.

### Strengths

To our knowledge, we are the first to report meta-analyses on the educational impact of Mini-CEX and DOPS. By applying a sensitive and broad search strategy, including undergraduate and postgraduate medical trainees as well as trainees from health professions other than human medicine, we assume that we detected all relevant articles on the educational impact of Mini-CEX and DOPS. To analyze potential influences on the educational impact of Mini-CEX and DOPS, we applied a framework of implementation characteristics according to Durlak and DuPre [17].

### Limitations

Despite our comprehensive search strategy, we found only four articles which reported effects of Mini-CEX/DOPS on Kirkpatrick level 2. Whether the positive effects on undergraduate trainees also hold true in postgraduate settings needs to be investigated. Although we analyzed eleven potential influences on the educational impact of Mini-CEX and DOPS, we found only two to be significantly associated with the educational impact of Mini-CEX. Due to the limited data basis, we might have underestimated the number of associations of the educational impact of Mini-CEX and DOPS with characteristics of the setting and of the implementation status.

### Conclusions

Despite the limited evidence regarding the educational impact of Mini-CEX and DOPS as well as the limited reports on implementation status, we demonstrated positive effects of Mini-CEX and DOPS on trainee performance in meta-analyses and identified two implementation characteristics to be associated with the educational impact of Mini-CEX. The positive results of the meta-analyses might help educators to justify and strengthen the use of these tools in health professions training. The finding that two characteristics of the implementation status are associated with the educational impact puts further emphasis on the proper implementation of Mini-CEX and DOPS in order to increase their educational impact.

## Supporting information

S1 Table PRISMA 2009 checklist. (PDF)

S2 Table Definitions of potential influences and their classifications. (PDF)

S3 Table Studies included in systematic review and extracted data. Classification of extracted data is displayed in italics. Abbreviations: ACE (assessment of clinical expertise), CbD (case based discussion), CP (case conference), FY (foundation year), ITER (in-training evaluation reports), JCP (journal club presentation), Mini-ACE (mini-assessed clinical encounter), Mini-PAT (mini-peer assessment tool), MSF (multisource feedback), OSCE (objective structured clinical examination), OSLER (objective structured long examination record), PDA (personal digital assistant), PSQ (patient satisfaction questionnaire), WPBA (workplace-based assessment).
(PDF)
