# Supplementary material for: The educational impact of Mini-Clinical Evaluation Exercise (Mini-CEX) and Direct Observation of Procedural Skills (DOPS) and its association with implementation: A systematic review and meta-analysis
Source: PLoS One. 2018 Jun 4;13(6):e0198009. doi: 10.1371/journal.pone.0198009 (PMC5986126; doi:10.1371/journal.pone.0198009)
Supplement: S2 Table — (PDF) [file pone.0198009.s002.pdf]

Characteristic of the setting	Definition	Category
Purpose of Mini-CEX/DOPS	Purpose of Mini-CEX/DOPS describes the purpose for which the tools were used.	Formative
		Formative and summative
		Summative
Mandatory Mini-CEX/DOPS	Mandatory Mini-CEX/DOPS describes whether the use of the tools was mandatory.	Yes: mandatory use of Mini-CEX/DOPS
		No: voluntary use of Mini-CEX/DOPS
Assessment sheet	Assessment sheet describes what the assessment sheet looked like regarding the use of scales and space for narrative comments.	Scales only
		Combination of scales and space for narrative comments
**Implementation characteristic**	**Definition**	**Classification**
Fidelity	Fidelity describes the extent to which Mini-CEX/DOPS was implemented as intended, especially regarding the purpose.	Low: the purpose of Mini-CEX/DOPS was not carried out as originally intended
		Medium: the purpose was partly caried out as intended
		High: the purpose of Mini-CEX/DOPS was carried out as intended
Dosage I	Dosage I describes the percentage of how many of the originally intended number of Mini-CEX/DOPS were delivered.	Low: less than one third of the originally intended number of Mini-CEX/DOPS was performed
		Medium: between one and two thirds of the originally intended number of Mini-CEX/DOPS were performed
		High: more than two thirds of the originally intended number of Mini-CX/DOPS were performed
Dosage II	Dosage II describes the mean number of actually performed Mini-CEX/DOPS per trainee and week.	Low: Mini-CEX/DOPS were performed less than four times a year
		Medium: Mini-CEX/DOPS were performed between four times a year and once every two weeks
		High: Mini-CEX/DOPS were performed more than once every two weeks

Quality	Quality describes whether and how well direct observation, assessment, and feedback were conducted.	Low: Mini-CEX/DOPS were not performed properly, trainees altered their behavior during direct observation, or feedback quality was poor
		Medium: Mini-CEX/DOPS were partly not performed properly, part of the trainees altered their behavior during direct observation, feedback quality was partly poor
		High: Mini-CEX/DOPS were performed properly, trainees did not alter their performance during direct observation, feedback quality was high
Participant responsiveness	Participant responsiveness describes the degree to which the program stimulated the interest or held the attention of participants.	Low: trainees and supervisors did not find Mini-CEX/DOPS helpful
		Medium: some trainees and supervisors found Mini-CEX/DOPS helpful, some did not
		High: trainees and supervisors found Mini-CEX/DOPS helpful
Program differentiation	Program differentiation describes the extent to which Mini-CEX/DOPS can be distinguished from other programs such as in-training evaluation reports, case based discussion, or multisource feedback.	Low: other workplace-based assessment tools besides Mini-CEX/DOPS
		Medium: in-training evaluation reports besides Mini-CEX/DOPS
		High: no similar tools to Mini-CEX/DOPS
Monitoring of control	Monitoring of control describes whether the nature and amount of direct observation and feedback received by the control group was reported.	Yes: the nature and amount of direct observation and feedback received by the control group was reported
		No: the nature and amount of direct observation and feedback received by the control group was not reported
Program reach	Program reach describes the rate of trainees that actually received Mini-CEX/DOPS.	Low: less than one third of the trainees received Mini-CEX/DOPS
		Medium: between one and two thirds of the trainees received Mini-CEX/DOPS
		High: more than two thirds of the trainees received Mini-CEX/DOPS
Adaptation	Adaptation describes whether changes were made in the original program during implementation.	Yes: adaptations were made during implementation
		No: no adaptations were made during implementation
Educational impact	Educational impact was operationalized according to Barr's adaptation of Kirkpatrick's four-level model.	Low: trainees did not find Mini-CEX/DOPS helpful or Mini-CEX/DOPS had a negative impact on trainee performance
		Medium: satisfaction with Mini-CEX/DOPS was mixed or the tools had no impact on trainee performance
		High: Mini-CEX/DOPS was perceived as helpful or had a positive impact on trainee performance

Supplement 2: Definitions of potential influences and their classifications.
