# Supplementary material for: The educational impact of Mini-Clinical Evaluation Exercise (Mini-CEX) and Direct Observation of Procedural Skills (DOPS) and its association with implementation: A systematic review and meta-analysis
Source: PLoS One. 2018 Jun 4;13(6):e0198009. doi: 10.1371/journal.pone.0198009 (PMC5986126; doi:10.1371/journal.pone.0198009)
Supplement: S3 Table — Classification of extracted data is displayed in italics. Abbreviations: ACE (assessment of clinical expertise), CbD (case based discussion), CP (case conference), FY (foundation year), ITER (in-training evaluation reports), JCP (journal club presentation), Mini-ACE (mini-assessed clinical encounter), Mini-PAT (mini-peer assessment tool), MSF (multisource feedback), OSCE (objective structured clinical examination), OSLER (objective structured long examination record), PDA (personal digital assistant), PSQ (patient satisfaction questionnaire), WPBA (workplace-based assessment). (PDF) [file pone.0198009.s003.pdf]

Kirkpatrick level 1
Mini-CEX

Study (citation number)	Setting		Study design		Implementation status		Reported outcome: educational impact of WPBA	Kirkpatrick level	Interpretation of educational impact	MERSQI
Lau Yanting et al., 2016 (34)	Institution	National University of Singapore Yong Loo Lin, School of Medicine and seven major clinical teaching hospitals, Singapore	Aim of study	To assess experiences with Mini-CEX	Fidelity	Not stated	81.4 % agreed or strongly agreed with the statement: "I feel that the following method of assessment is effective for medical undergraduates for learning in advanced years, or to prepare myself for future practice as a doctor."	Level 1	Mini-CEX is perceived as helpful for learning *(high)*	6.5
	Mini-CEX/DOPS	Mini-CEX	Method	Descriptive study using questionnaire	Dosage	Not applicable				
	Purpose of Mini-CEX/DOPS	Formative and summative	Intervention	Formative and summative Mini-CEX	Quality	Strong inter-rater variability				
	Feedback recipient	All students from year 3 to 5	Control	None	Participant responsiveness	Not stated				
	Mandatory Mini-CEX/DOPS	Yes	Sample	275 undergraduate medical trainees	Program differentiation	High				
	Number of Mini-CEX/DOPS	Not stated			Monitoring of control	Not applicable				
	Assessment sheet	Not stated			Program reach	100% *(high)*				
	Feedback provider	Clinical tutors			Adaptation	None				
	Similar tools	No								
Vaughan and Moore, 2016 (40)	Institution	Victoria University Osteopathy Clinic, Melbourne, Australia	Aim of study	To provide validity evidence for Mini-CEX	Fidelity	Not stated	Trainees' satisfaction with Mini-CEX was 4.75 on a 6-point scale	Level 1	High satisfaction with Mini-CEX *(high)*	7
	Mini-CEX/DOPS	Mini-CEX	Method	Descriptive study using overall satisfaction rating within Mini-CEX	Dosage	87% *(Dosage I: high)* resulting in 0.07 Mini-CEX per week *(Dosage II: low)*				
	Purpose of Mini-CEX/DOPS	Formative	Intervention	Formative Mini-CEX	Quality	Not stated				
	Feedback recipient	First-year osteopathy Master students during sessions in the Victoria University Osteopathy Clinic	Control	None	Participant responsiveness	Trainees were satisfied with Mini-CEX *(high)*				
	Mandatory Mini-CEX/DOPS	Yes	Sample	96 undergraduate osteopathy trainees	Program differentiation	Not applicable				
	Number of Mini-CEX/DOPS	At least 4 Mini-CEX per year			Monitoring of control	Not applicable				
	Assessment sheet	6 sub-scales, one global rating, space for narrative comments regarding stregnth, area for improvement and action plan			Program reach	100%				
	Feedback provider	Clinical educators (registered osteopath and another registered health professional involved in student supervision)			Adaptation	Not stated				
	Similar tools	Not stated								

Castanelli et al., 2016 (30)	Institution	Australian and New Zealand College of Anaesthetists, Australia and New Zealand	Aim of study	To explore experiences with Mini-CEX	Fidelity	The purpose of the Mini-CEX was not always clear to the trainees *(medium)*	Some trainees reported benefits that the mini-CEX brings to their learning	Level 1	Mini-CEX is perceived as rather helpful for medical training *(medium)*	6.5
	Mini-CEX/DOPS	Mini-CEX	Method	Qualitative study using semi-structured telephone interviews	Dosage	Not applicable				
	Purpose of Mini-CEX/DOPS	Formative and summative	Intervention	Formative and summative Mini-CEX	Quality	Inter-rater variability				
	Feedback recipient	Anaesthesia trainees	Control	None	Participant responsiveness	Some trainees reported benefits that Mini-CEX brings to their learning. *(medium)*				
	Mandatory Mini-CEX/DOPS	Yes	Sample	17 postgraduate medical trainees	Program differentiation	Low				
	Number of Mini-CEX/DOPS	Not stated			Monitoring of control	Not applicable				
	Assessment sheet	13 sub-scales, 1 global rating, space for narrative comments			Program reach	Not stated				
	Feedback provider	Specialist anaesthetists acting as supervisors			Adaptation	Yes: Some supervisors broadened the scope of Mini-CEX and included non-technical skills and other				
	Similar tools	Other WPBAs								
Saeed et al., 2015 (37)	Institution	Shifa College of Medicine, Islamabad, Pakistan	Aim of study	To assess satisfaction with Mini-CEX	Fidelity	Not stated	Satisfaction with Mini-CEX was 8.0 on a 9/10-point scale (scale was reported inconsistently).	Level 1	High satisfaction with Mini-CEX *(high)*	6
	Mini-CEX/DOPS	Mini-CEX	Method	Descriptive study using overall satisfaction rating directly after each Mini-CEX	Dosage	98% *(Dosage I: high)* resulting in 0.19 Mini-CEX/week *(Dosage II: medium)*				
	Purpose of Mini-CEX/DOPS	Formative	Intervention	Formative Mini-CEX	Quality	Not stated				
	Feedback recipient	4th-year students in 5 clinical rotations of two months each (medicine, surgery, obstestric/gynecology/pediatrics, ophthalmology/ENT, family medicine/community medicine)	Control	None	Participant responsiveness	Students' and supervisors' satisfaction with Mini-CEX was high. *(high)*				
	Mandatory Mini-CEX/DOPS	Not stated	Sample	96 undergraduate medical trainees	Program differentiation	Not applicable				
	Number of Mini-CEX/DOPS	10 Mini-CEX in one year (2 Mini-CEX per rotation)			Monitoring of control	Not applicable				
	Assessment sheet	6 sub-scales, 1 global rating, and space for written feedback			Program reach	All 4th-year students in their 5 clinical rotations *(high)*				
	Feedback provider	Senior registars, assistant professors, associate porfessors and consultants			Adaptation	Not stated				
	Similar tools	Not stated								

Weijs et al, 2015 (48)	Institution	Ontario Veterinary College and Hill's Pet Nutrition Primary Health Centre, Canada	Aim of study	To evaluate feasibility, acceptability, and learning impact of Mini-CEX	Fidelity	Not stated	Trainees felt that Mini-CEX was useful and valuable for trainees' learning	Level 1	Mini-CEX is perceived as useful *(high)*	7
	Mini-CEX/DOPS	Mini-CEX and DOPS	Method	Qualitative study using focus groups	Dosage	Not stated				
	Purpose of Mini-CEX/DOPS	Formative	Intervention	Formative Mini-CEX	Quality	Inter-rater variability				
	Feedback recipient	All final-year veterinary students in small animal rotation	Control	None	Participant responsiveness	Trainees and supervisors felt that Mini-CEX was useful and valuable for trainees' learning *(high)*				
	Mandatory Mini-CEX/DOPS	Yes	Sample	60 undergraduate veterinary trainees	Program differentiation	Low				
	Number of Mini-CEX/DOPS	At least 2 Mini-CEX and 2 DOPS within 3 weeks			Monitoring of control	Not applicable				
	Assessment sheet	Includes sub-scales, global rating and space for narrative comments			Program reach	100% *(high)*				
	Feedback provider	Clinical instructors			Adaptation	Not stated				
	Similar tools	ITER								
Weston et al., 2014 (26)	Institution	Northwest Thames Foundation School, Imperial College London, UK	Aim of study	To investigate current experiences, opinions and attitudes towards mini-CEX	Fidelity	Supervisors seemed not to understand how to use Mini-CEX. Tools were carried out only because of mandatory requirement (not as tool for learning). Mini-CEX remained a tick box exercise. *(low)*	"Do you find the mini-CEX a useful part of your training as an FY1 doctor?" was rated 2.5 on a 5-point scale (1-5). "Do you feel that the use of the mini-CEX helps to bring about improvement in your practice as an FY1 doctor?" was rated 2.5 on a 5-point scale (1-5).	Level 1	Mini-CEX is rather not perceived as helpful for medical training (low)	7
	Mini-CEX/DOPS	Mini-CEX	Method	Descriptive study using questionnaire	Dosage	Not applicable				
	Purpose of Mini-CEX/DOPS	Formative	Intervention	Formative Mini-CEX	Quality	Often Mini-CEX was not completed properly. Constructive feedback and an action plan were not always included. *(low)*				
	Feedback recipient	FY1 doctors	Control	None	Participant responsiveness	Trainees found Mini-CEX rather not useful for training. However, Mini-CEX was perceived as a good idea. Supervisors' attitudes toward the assessment seemed poor and non-constructive (as perceived by the trainees). Trainees who had received training found the Mini-CEX more helpful than untrained trainees. *(low)*				
	Mandatory Mini-CEX/DOPS	Yes	Sample	50 postgraduate medical trainees	Program differentiation	Low				
	Number of Mini-CEX/DOPS	Not stated			Monitoring of control	Not applicable				
	Assessment sheet	Scales, space for action plan			Program reach	Not stated				
	Feedback provider	A variety of supervisors ranging from early specialists to consultants			Adaptation	Not stated				
	Similar tools	Other WPBAs								

Behere, 2014 (7)	Institution	Department of Oral Medicine and Radiology at STES's Sinhgad Dental College and Hospital, Pune, India	Aim of study	To implement and evaluate Mini-CEX	Fidelity	Not stated	All trainees felt that the constructive feedback helped reinforce the skills that they did well, and helped them identify weak areas.	Level 1	Mini-CEX is perceived as useful *(high)*	7
	Mini-CEX/DOPS	Mini-CEX	Method	Evaluation study using questionnaire	Dosage	100% *(Dosage I: high)* resulting in 1 Mini-CEX *(Dosage II: not applicable)*				
	Purpose of Mini-CEX/DOPS	Formative	Intervention	Formative Mini-CEX	Quality	All trainees received structured and elaborate feedback *(high)*				
	Feedback recipient	12 4th-year dental Bachelor students	Control	None	Participant responsiveness	Trainees appreciated the Mini-CEX, especially the feedback provided. Some felt that the presence of a teacher was intimidating. Supervisors found Mini-CEX time-consuming, but appreciated the tool. *(high)*				
	Mandatory Mini-CEX/DOPS	Yes	Sample	12 undergraduate dental trainees	Program differentiation	Not applicable				
	Number of Mini-CEX/DOPS	1 Mini-CEX			Monitoring of control	Not applicable				
	Assessment sheet	6 sub-scales, one global rating, space for narrative comments regarding stregnth, area for improvement and action plan			Program reach	100% *(high)*				
	Feedback provider	4 teachers			Adaptation	Not stated				
	Similar tools	Not stated								
Sweet et al., 2013 (8)	Institution	Australia	Aim of study	To develop, implement and evaluate a Mini-CEX tool for midwifery education	Fidelity	Not stated	Trainees were very positive about the value of the midwifery Mini-CEX for enhancing their learning	Level 1	Mini-CEX is perceived as very useful *(high)*	7
	Mini-CEX/DOPS	Mini-CEX	Method	Implementation study using focus groups	Dosage	Not applicable				
	Purpose of Mini-CEX/DOPS	Formative	Intervention	Formative Mini-CEX	Quality	Not stated				
	Feedback recipient	2nd- and 3rd-year Bachelor midwifery students in Australia	Control	None	Participant responsiveness	Midwifery staff expressed enthusiasm about the tool and its potential application beyond the Bachelor of Midwifery students. The midwifery students were very positive about the applicability and value of the midwifery Mini-CEX for enhancing their learning. *(high)*				
	Mandatory Mini-CEX/DOPS	No	Sample	13 undergraduate midwifery trainees	Program differentiation	High				
	Number of Mini-CEX/DOPS	Not stated			Monitoring of control	Not applicable				
	Assessment sheet	6 sub-scales, one global rating, space for narrative comments regarding stregnth, area for improvement and action plan			Program reach	87% *(high)*				
	Feedback provider	Experienced nurses			Adaptation	Not stated				
	Similar tools	None								

Brazil et al., 2012 (30)	Institution	Royal Brisbane and Women's Hospital, Queensland, Australia	Aim of study	To assess satisfaction with Mini-CEX and its perceived educational value	Fidelity	Not stated	Rating: High overall satisfaction among interns.<br><br>Survey: Trainees felt that the formative impact of the mini-CEX was significant, and that it facilitated timely and specific feedback.	Level 1	High satisfaction with Mini-CEX *(high)*	7
	Mini-CEX/DOPS	Mini-CEX	Method	Implementation study using overall satisfaction rating directly after each Mini-CEX and questionnaire	Dosage	95% *(Dosage I: high)* resulting in 0.76 Mini-CEX/week *(Dosage II: high)*				
	Purpose of Mini-CEX/DOPS	Formative and summative	Intervention	Formative and summative Mini-CEX	Quality	Not stated				
	Feedback recipient	20 term 4 interns in the emergency department	Control	None	Participant responsiveness	Trainees appreciated the timely and specific feedback *(high)*				
	Mandatory Mini-CEX/DOPS	Yes	Sample	19 postgraduate medical trainees	Program differentiation	Medium				
	Number of Mini-CEX/DOPS	4 Mini-CEX in 5 weeks			Monitoring of control	Not applicable				
	Assessment sheet	6 sub-scales, 1 global rating; space for narrative comments regarding trainees' strengths and suggestions for development			Program reach	All 20 interns in the emergency department received Mini-CEX *(high)*				
	Feedback provider	7 emergency department consultants and one senior registrar			Adaptation	Not stated				
	Similar tools	ITER								
Sabey et al., 2011 (28)	Institution	Two training locations with Severn Deanery, UK	Aim of study	To evaluate the implementation of Mini-CEX	Fidelity	Not stated	10% rated Mini-CEX as 'useful' or 'very useful'	Level 1	Mini-CEX is not perceived as useful *(low)*	6.5
	Mini-CEX/DOPS	Mini-CEX and DOPS	Method	Implementation study using questionnaires and focus groups/semi-structured interviews	Dosage	Not applicable				
	Purpose of Mini-CEX/DOPS	Not stated	Intervention	Mini-CEX (purpose not stated)	Quality	Trainees were doubtful about the level of honesty of feedback received in Mini-CEX *(low)*				
	Feedback recipient	General practice trainees	Control	None	Participant responsiveness	Few trainees perceived Mini-CEX as useful *(low)*				
	Mandatory Mini-CEX/DOPS	Yes	Sample	74 postgraduate medical trainees (52 answered the questionnaire; 22 participated in focus groups/interviews)	Program differentiation	low				
	Number of Mini-CEX/DOPS	Not stated			Monitoring of control	Not applicable				
	Assessment sheet	Not stated			Program reach	Not stated				
	Feedback provider	Not stated			Adaptation	Not stated				
	Similar tools	MSF, CbD								

Bindal et al., 2011 (47)	Institution	West Midlands Deanery, UK	Aim of study	To evaluate the implementation of Mini-CEX	Fidelity	Not stated	"How useful are workplace-based assessments for helping with your medical training?" was rated 3.2 for Mini-CEX on a 6-point scale (1-6).	Level 1	Mini-CEX is rather not perceived as helpful *(medium)*	8
	Mini-CEX/DOPS	Mini-CEX and DOPS	Method	Implementation study using questionnaire	Dosage	Not applicable				
	Purpose of Mini-CEX/DOPS	Not stated	Intervention	Mini-CEX (purpose not stated)	Quality	Not stated				
	Feedback recipient	Paediatric trainees	Control	None	Participant responsiveness	Mini-CEX and DOPS are rather not perceived as helpful *(low)*				
	Mandatory Mini-CEX/DOPS	Yes	Sample	130 postgraduate medical trainees	Program differentiation	low				
	Number of Mini-CEX/DOPS	Not stated			Monitoring of control	Not applicable				
	Assessment sheet	Modified for paediatric training			Program reach	Not stated				
	Feedback provider	Senior trainees or consultants			Adaptation	Not stated				
	Similar tools	MSF, CbD								
Jackson and Wall, 2010 (27)	Institution	Not stated, UK	Aim of study	To evaluate trainees' satisfaction with and the educational impact of Mini-CEX	Fidelity	Not stated	Satisfaction with Mini-CEX was rated 3.9 on a 10-point scale (1-10)	Level 1	Low satisfaction with Mini-CEX *(low)*	8
	Mini-CEX/DOPS	Mini-CEX	Method	Descriptive study using focus groups and questionnaires	Dosage	Not stated				
	Purpose of Mini-CEX/DOPS	Not stated	Intervention	Mini-CEX (purpose not stated)	Quality	Poor (direct observation and/or feedback conversation were frequently not performed), 60% of trainees felt that the supervisors did not know what they were doing during a mini-CEX encounter *(low)*				
	Feedback recipient	FY1 doctors	Control	None	Participant responsiveness	Participants felt that the Mini-CEX was an unrealistic reflection of their performance. Low satisfaction with the Mini-CEX *(low)*				
	Mandatory Mini-CEX/DOPS	Yes	Sample	45 postgraduate medical trainees (8 participated in focus groups; 37 answered the questionnaire)	Program differentiation	Not applicable				
	Number of Mini-CEX/DOPS	6 Mini-CEX per year			Monitoring of control	Not applicable				
	Assessment sheet	Not stated			Program reach	Not stated				
	Feedback provider	Not stated			Adaptation	Not stated				
	Similar tools	Not stated								

Weller, Jolly et al., 2009 (41)	Institution	Auckland City Hospital, New Zealand	Aim of study	To evaluate the use of the Mini-CEX and its impact on the quality and quantity of feedback	Fidelity	Not stated	Mean satisfaction with the use of Mini-CEX was 7.3 on a 10-point scale.<br><br>72% felt that Mini-CEX improved the frequency of feedback.<br>83% felt that Mini-CEX improved the quality of feedback.<br>48% felt that Mini-CEX improved their confidence in asking for feedback.	Level 1	High satisfaction with Mini-CEX *(high)*	7
	Mini-CEX/DOPS	Mini-CEX	Method	Descriptive study using questionnaire	Dosage	Not applicable				
	Purpose of Mini-CEX/DOPS	Formative	Intervention	Formative Mini-CEX	Quality	95% of specialists commented on trainees' strengths, 70% on trainees' weaknesses, 60% defined an action plan *(high)*				
	Feedback recipient	Anaesthesia trainees	Control	None	Participant responsiveness	Specialists and trainees agreed that the instrument improved the frequency and quality of feedback *(high)*				
	Mandatory Mini-CEX/DOPS	No	Sample	30 postgraduate medical trainees	Program differentiation	Not applicable				
	Number of Mini-CEX/DOPS	Not stated			Monitoring of control	Not applicable				
	Assessment sheet	Online form; adapted for anaesthesia; includes scales and space for narrative comments regarding trainees' strengths, weaknesses and action plan			Program reach	Not stated				
	Feedback provider	Specialists			Adaptation	Not stated				
	Similar tools	Not stated								
Weller, Jones, et al., 2009 (42)	Institution	5 anaesthetic departments in Australia	Aim of study	To analyze experience with the Mini-CEX	Fidelity	Not stated	Trainees appreciated being assessed. Trainees felt that the Mini-CEX facilitated feedback.	Level 1	Moderate satisfaction with Mini-CEX *(medium)*	7
	Mini-CEX/DOPS	Mini-CEX	Method	Descriptive study using surveys and focus groups	Dosage	Not applicable				
	Purpose of Mini-CEX/DOPS	Formative	Intervention	Formative Mini-CEX	Quality	Some trainees altered their behaviour during the assessments; inter-rater variability; partly poor feedback quality *(medium)*				
	Feedback recipient	Anaesthesia trainees	Control	None	Participant responsiveness	Mini-CEX was not perceived as a good instrument to measure own progress. Mini-CEX was perceived useful for learning. Trainees appreciated the assessments. Specialists found giving feedback easier with the Mini-CEX. *(medium)*				
	Mandatory Mini-CEX/DOPS	No	Sample	40 postgraduate medical trainees (29 answered the survey; 11 participated in focus groups)	Program differentiation	Medium				
	Number of Mini-CEX/DOPS	Not stated			Monitoring of control	Not applicable				
	Assessment sheet	Modified online version; 9 sub-scales, 1 global rating; space for narrative comments regarding trainees' strengths, weaknesses and action plan			Program reach	Not stated				
	Feedback provider	Specialists			Adaptation	Not stated				
	Similar tools	ITER								

Malhotra et al., 2008 (35)	Institution	University of British Columbia, Canada	Aim of study	To investigate trainees' perception of Mini-CEX	Fidelity	Not stated	Many residents found the mini-CEX to be a useful learning tool.	Level 1	Mini-CEX is perceived as useful *(high)*	6
	Mini-CEX/DOPS	Mini-CEX	Method	Qualitative study using focus groups	Dosage	Not stated				
	Purpose of Mini-CEX/DOPS	Formative	Intervention	Formative Mini-CEX	Quality	Not stated				
	Feedback recipient	Internal medicine residents	Control	None	Participant responsiveness	The residents' perception was influenced by anxiety. They perceived a dualism between assessment and education (summative and formative assessment). When residents had more experience with the format, they became more comfortable with it and found the Mini-CEX a useful learning tool. *(medium)*				
	Mandatory Mini-CEX/DOPS	Yes	Sample	12 postgraduate medical trainees	Program differentiation	medium				
	Number of Mini-CEX/DOPS	At least 6 Mini-CEX per year (1 per rotation)			Monitoring of control	Not applicable				
	Assessment sheet	Not stated			Program reach	Not stated				
	Feedback provider	Not stated			Adaptation	Not stated				
	Similar tools	ITER								
Nair et al., 2008 (36)	Institution	Three large metropolitan teaching hospitals in New South Wales, Queensland, and Victoria, Australia	Aim of study	To evaluate acceptability of the Mini-CEX	Fidelity	Not stated	Almost half of the trainees (7/16) were satisfied or very satisfied with the mini-CEX as a tool for learning.	Level 1	Medium satisfaction with Mini-CEX *(medium)*	8
	Mini-CEX/DOPS	Mini-CEX	Method	Descriptive study using questionnaire	Dosage	75% *(Dosage I: high)* resulting in 0.14 Mini-CEX/week *(Dosage II: medium)*				
	Purpose of Mini-CEX/DOPS	Formative	Intervention	Formative Mini-CEX	Quality	75% received feedback after each Mini-CEX, of which 75% were satisfied with this feedback *(high)*				
	Feedback recipient	28 International medical graduates	Control	None	Participant responsiveness	Almost half of the trainees were satisfied or very satisfied with the Mini-CEX as a tool for learning *(medium)*				
	Mandatory Mini-CEX/DOPS	No	Sample	16 postgraduate medical trainees	Program differentiation	Not applicable				
	Number of Mini-CEX/DOPS	10 Mini-CEX (4 Mini-CEX in emergency medicine, 3 in medicine, and 3 in surgery) within 1 year			Monitoring of control	Not applicable				
	Assessment sheet	6 sub-scales; 1 global rating			Program reach	100% *(high)*				
	Feedback provider	Specialists and specialist trainees in internal medicine, surgery and emergency medicine			Adaptation	Not stated				
	Similar tools	Not stated								

De Lima et al., 2007 (32)	Institution	Several institutions affiliated to the University of Buenos Aires, Argentina	Aim of study	To determine the validity, reliability, feasibility and satisfaction of the Mini-CEX	Fidelity	Not stated	Trainees' mean satisfaction with Mini-CEX was 8.1 on a nine-point scale	Level 1	High satisfaction with Mini-CEX *(high)*	8
	Mini-CEX/DOPS	Mini-CEX	Method	Descriptive study using satisfaction rating on the Mini-CEX sheet	Dosage	About 60% *(Dosage I: medium)* resulting in 0.03 Mini-CEX/week *(Dosage II: low)*				
	Purpose of Mini-CEX/DOPS	Formative	Intervention	Formative Mini-CEX	Quality	Not stated				
	Feedback recipient	Cardiology residents from 17 cardiology training programs	Control	None	Participant responsiveness	High satisfaction with Mini-CEX *(high)*				
	Mandatory Mini-CEX/DOPS	Yes	Sample	108 postgraduate medical trainees	Program differentiation	High				
	Number of Mini-CEX/DOPS	At least 4 encounters within 19 months			Monitoring of control	Not applicable				
	Assessment sheet	6 sub-scales; 1 global rating			Program reach	Not stated				
	Feedback provider	Faculty members			Adaptation	Not stated				
	Similar tools	None								
Torre et al., 2007 (39)	Institution	Medical College of Wisconsin and at different clinical teaching sites or outpatient sites, USA	Aim of study	To determine the feasibility, implementation, and user satisfaction with a PDA-based Mini-CEX	Fidelity	Not stated	Trainees satisfaction with the PDA-based Mini-CEX was 8.0 on a nine-point scale	Level 1	High satisfaction with PDA-based Mini-CEX *(high)*	8
	Mini-CEX/DOPS	PDA-based Mini-CEX	Method	Descriptive study using satisfaction rating within the PDA-based Mini-CEX	Dosage	100% *(Dosage I: high)* resulting in 0.22 Mini-CEX/week *(Dosage II: medium)*				
	Purpose of Mini-CEX/DOPS	Formative	Intervention	formative PDA-based Mini-CEX	Quality	Feedback was provided in 96% of PDA-based Mini-CEX *(high)*				
	Feedback recipient	All 3rd-year medical students in their 2 month core clinical clerkship in internal medicine	Control	None	Participant responsiveness	High satisfaction with the tool among trainees and supervisors *(high)*				
	Mandatory Mini-CEX/DOPS	Yes	Sample	117 undergraduate medical trainees	Program differentiation	High				
	Number of Mini-CEX/DOPS	2 Mini-CEX (1 per month)			Monitoring of control	Not applicable				
	Assessment sheet	Adaptation of paper-based Mini-CEX to PDA; 6 sub-scales; 1 global rating			Program reach	100% *(high)*				
	Feedback provider	Senior residents and faculty members			Adaptation	Not stated				
	Similar tools	None								

Kirkpatrick level 1<br>DOPS										
**Study (citation number)**	**Setting**		**Study design**		**Implementation status**		**Reported outcome: educational impact of WPBA**	**Kirkpatrick level**	**Interpretation of educational impact**	**MERSQI**
Weijs et al., 2015 (48)	Institution	Ontario Veterinary College and Hill's Pet Nutrition Primary Health Centre, Canada	Aim of study	To evaluate feasibility, acceptability, and learning impact of DOPS	Fidelity	Not stated	Trainees felt that DOPS was valuable for their learning	Level 1	DOPS is perceived as useful *(high)*	7
	Mini-CEX/DOPS	Mini-CEX and DOPS	Method	Qualitative study using focus groups	Dosage	Not stated				
	Purpose of Mini-CEX/DOPS	Formative	Intervention	Formative DOPS	Quality	Inter-rater variability				
	Feedback recipient	All final-year veterinary students in small animal rotation	Control	None	Participant responsiveness	Trainees and supervisors felt that DOPS was valuable for trainees' learning *(high)*				
	Mandatory Mini-CEX/DOPS	Yes	Sample	60 undergraduate veterinary trainees	Program differentiation	Low				
	Number of Mini-CEX/DOPS	At least 2 Mini-CEX and 2 DOPS within 3 weeks			Monitoring of control	Not applicable				
	Assessment sheet	Includes sub-scales, global rating and space for narrative comments			Program reach	100% *(high)*				
	Feedback provider	Clinical instructors			Adaptation	Not stated				
	Similar tools	ITER								
Hoseini et al., 2013 (45)	Institution	University hospitals affiliated to Mashhad University of Medical Sciences, Iran	Aim of study	To assess satisfaction with DOPS and compare it to satisfaction with logbook	Fidelity	Not stated	Satisfaction mean score of midwifery trainees with DOPS was 76.7±12.9 (out of 100). Satisfaction with DOPS was significantly higher than with logbook (t-test, p<0.000) score obtained in current method (P < 0.000).	Level 1	High satisfaction with DOPS *(high)*	9
	Mini-CEX/DOPS	DOPS	Method	Controlled trial; Outcome: satisfaction rating	Dosage	Not stated				
	Purpose of Mini-CEX/DOPS	Formative	Intervention	Formative DOPS	Quality	Not stated				
	Feedback recipient	Midwifery students	Control	Logbook consisting of four parts: 1) a documentation form for practicing skills by students; 2) a documentation and evaluation form for scientific discussions held by students; 3) a documentation form for management of high-risk maternity cases; and 4) a student evaluation form filled out by the instructors.	Participant responsiveness	DOPS was perceived as very useful in learning and enhancing clinical skills. *(high)*				
	Mandatory Mini-CEX/DOPS	Not stated	Sample	Intervention: 33 undergraduate midwifery trainees; Control: 34 undergraduate midwifery trainees	Program differentiation	Not applicable				
	Number of Mini-CEX/DOPS	Not stated			Monitoring of control	Yes				
	Assessment sheet	Not stated			Program reach	100% *(high)*				
	Feedback provider	Clinical supervisor			Adaptation	Not stated				
	Similar tools	Not stated								

Brittlebank et al., 2013 (43)	Institution	Royal College of Psychiatrists, 16 sites across England, Scotland and Wales	Aim of study	To assess satisfaction with DOPS	Fidelity	Not stated	Trainee satisfaction with DOPS was 4.8 on a 6-point scale	Level 1	Moderate satisfaction with DOPS *(medium)*	6
	Mini-CEX/DOPS	DOPS	Method	Descriptive study using overall satisfaction rating	Dosage	Not applicable				
	Purpose of Mini-CEX/DOPS	Not stated	Intervention	DOPS (purpose not stated)	Quality	Not stated				
	Feedback recipient	600 psychiatry trainees in the first 3 years of specialty training	Control	None	Participant responsiveness	Trainee satisfaction with DOPS was 4.8 on a 6-point scale *(medium)*				
	Mandatory Mini-CEX/DOPS	No	Sample	133 postgraduate medical trainees	Program differentiation	low				
	Number of Mini-CEX/DOPS	Not stated			Monitoring of control	Not applicable				
	Assessment sheet	Not stated			Program reach	Not stated				
	Feedback provider	Not stated			Adaptation	Not stated				
	Similar tools	CbD, ACE, Mini-ACE, Mini-PAT, PSQ, CP, JCP								
Bindal et al., 2013 (47)	Institution	Birmingham School of Anaesthesia (all anaesthetic departments), UK	Aim of study	To explore opinions and experiences with DOPS	Fidelity	Not stated	"DOPS are useful for training" was rated 2.7 on a 6-point scale (1-6)	Level 1	DOPS is rather not perceived as useful for training *(low)*	7
	Mini-CEX/DOPS	DOPS	Method	Implementation study using questionnaire	Dosage	Not stated				
	Purpose of Mini-CEX/DOPS	Not stated	Intervention	DOPS (purpose not stated)	Quality	The space for future improvements was often not completed				
	Feedback recipient	Anaesthesia trainees in their FYs and in specialist training in Birmingham	Control	None	Participant responsiveness	Trainees and consutants felt that DOPS did not reflect trainees' capabilities. Trainees and consultants did not find DOPS a helpful tool for training. *(low)*				
	Mandatory Mini-CEX/DOPS	Yes	Sample	90 postgraduate medical trainees	Program differentiation	Not applicable				
	Number of Mini-CEX/DOPS	Minimum of 75 DOPS in 7 years of anaesthetic training program			Monitoring of control	Not applicable				
	Assessment sheet	Space for future improvements			Program reach	Not stated				
	Feedback provider	Consultant staff			Adaptation	Not stated				
	Similar tools	Not stated								
Sabey et al, 2011 (28)	Institution	Two training locations with Severn Deanery, UK	Aim of study	To evaluate the implementation of DOPS	Fidelity	Not stated	14% rated DOPS as 'useful' or 'very useful'	Level 1	DOPS is not perceived as useful *(low)*	6.5
	Mini-CEX/DOPS	Mini-CEX and DOPS	Method	Implementation study using questionnaires and focus groups/semi-structured interviews	Dosage	Not applicable				
	Purpose of Mini-CEX/DOPS	Not stated	Intervention	DOPS (purpose not stated)	Quality	Often trainees were not observed during DOPS *(low)*				
	Feedback recipient	General practice trainees	Control	None	Participant responsiveness	Few trainees perceived DOPS as useful *(low)*				
	Mandatory Mini-CEX/DOPS	Yes	Sample	74 postgraduate medical trainees (52 answered the questionnaire; 22 participated in focus groups/interviews)	Program differentiation	low				
	Number of Mini-CEX/DOPS	Not stated			Monitoring of control	Not applicable				
	Assessment sheet	Not stated			Program reach	Not stated				
	Feedback provider	Not stated			Adaptation	Not stated				
	Similar tools	MSF, CbD								

Bindal et al., 2011 (47)	Institution	West Midlands Deanery, UK	Aim of study	To evaluate the implementation of DOPS	Fidelity	Not stated	"How useful are workplace-based assessments for helping with your medical training?" was rated 3.2 for DOPS on a 6-point scale (1-6).	Level 1	DOPS is rather not perceived as helpful *(medium)*	8
	Mini-CEX/DOPS	Mini-CEX and DOPS	Method	Implementation study using questionnaire	Dosage	Not applicable				
	Purpose of Mini-CEX/DOPS	Not stated	Intervention	DOPS (purpose not stated)	Quality	Not stated				
	Feedback recipient	Paediatric trainees	Control	None	Participant responsiveness	DOPS is rather not perceived as helpful *(low)*				
	Mandatory Mini-CEX/DOPS	Yes	Sample	130 postgraduate medical trainees	Program differentiation	low				
	Number of Mini-CEX/DOPS	Not stated			Monitoring of control	Not applicable				
	Assessment sheet	Modified for paediatric training			Program reach	Not stated				
	Feedback provider	Senior trainees or consultants			Adaptation	Not stated				
	Similar tools	MSF, CbD								
Morris et al., 2008 (46)	Institution	Whipps Cross Hospital University Trust, London, UK	Aim of study	To evaluate the implementation of DOPS	Fidelity	Not stated	10% found DOPS threatening. 75% found the presence of the clinical skill facilitator was useful. 70% thought of DOPS as a test. 70% thought DOPS would help improve their clinical skills. 65% thought that undertaking DOPs will improve their future career. 50% were aware of the DOPS assessment tool.	Level 1	DOPS is perceived as rather useful *(medium)*	7
	Mini-CEX/DOPS	Mini-CEX and DOPS	Method	Implementation study using questionnaire	Dosage	50% *(Dosage I: medium)* resulting in 0.04 DOPS/week *(Dosage II: low)*				
	Purpose of Mini-CEX/DOPS	Formative	Intervention	Formative DOPS	Quality	Not stated				
	Feedback recipient	27 FY1 residents	Control	None	Participant responsiveness	Initially, some trainees viewed DOPS as a test. This situation improved with time. Several residents made positive comments about the constructive feedback they received within DOPS. *(medium)*				
	Mandatory Mini-CEX/DOPS	Not stated	Sample	25 postgraduate medical trainees	Program differentiation	Low				
	Number of Mini-CEX/DOPS	4 DOPS; 1 Mini-CEX within 1 year			Monitoring of control	Not applicable				
	Assessment sheet	DOPS: preselected procedures, adapted checklist. Mini-CEX: not stated			Program reach	Not stated				
	Feedback provider	Consultant educational supervisor			Adaptation	Not stated				
	Similar tools	MSF								

Kirkpatrick level 2b<br>Mini-CEX										
**Study (citation number)**	**Setting**		**Study design**		**Implementation status**		**Reported outcome: educational impact of WPBA**	**Kirkpatrick level**	**Interpretation of educational impact**	**MERSQI**
Kim et al., 2016 (33)	Institution	Rutgers Robert Wood Johnson Medical School and clinical sites including one university and one to two community hospitals per clerkship, as well as multiple outpatient offices, USA	Aim of study	To assess the impact of mandatory Mini-CEX on trainee performance	Fidelity	Not stated	OSCE failure rate was significantly lower in intervention group (2%) compared to the control (12%)	Level 2b	The study suggests that mandatory Mini-CEX leads to improved clinical skills *(high)*	11.5
	Mini-CEX/DOPS	Mini-CEX	Method	Sequential cohort study; Outcome: failure rate in summative end-of-year objective structured clinical examination (OSCE)	Dosage	92% *(Dosage I: high)* resulting in 0.53 Mini-CEX/week *(Dosage II: high)*				
	Purpose of Mini-CEX/DOPS	Formative	Intervention	Mandatory formative Mini-CEX	Quality	The majority of forms indicated that specific feedback was given *(high)*				
	Feedback recipient	All 3rd-year students in the clerkships internal medicine, family medicine, pediatrics, psychiatry, obstetrics and gynecology, and surgery	Control	No or voluntary Mini-CEX	Participant responsiveness	Not stated				
	Mandatory Mini-CEX/DOPS	Yes	Sample	Intervention: 121 undergraduate medical trainees; Control: 114 undergraduate medical trainees	Program differentiation	Not applicable				
	Number of Mini-CEX/DOPS	23 Mini-CEX in 40 weeks			Monitoring of control	No				
	Assessment sheet	Modified Mini-CEX form, 7 sub-scales, space for suggestions for improvement			Program reach	Adherence to the mini-CEX requirement was high *(high)*				
	Feedback provider	Faculty and residents			Adaptation	Not stated				
	Similar tools	Not stated								

Suhoyo et al., 2014: Internal Medicine (38)	Institution	Faculty of Medicine, Universitas Gadjah Mada and teaching hospitals or affiliated hospitals, Indonesia	Aim of study	To investigate the effect of Mini-CEX on trainees' clinical competence	Fidelity	Not stated	Intervention group showed significantly higher OSLER results than control group (Mann–Whitney U test, Z=-2.34, p<0.05).	Level 2b	Positive effect of Mini-CEX on clinical performance *(high)*	12
	Mini-CEX/DOPS	Mini-CEX	Method	Sequential cohort study; Outcome: score in OSLER	Dosage	100% *(Dosage I: high)* resulting in 0.4 Mini-CEX/week *(Dosage II: medium)*				
	Purpose of Mini-CEX/DOPS	Formative and summative	Intervention	Formative and summative Mini-CEX	Quality	Not stated				
	Feedback recipient	Students in Internal Medicine clerkship	Control	Existing assessment program in clerkships including CbD, case reflections (more than in intervention group), logbooks, weekly supervisor feedback in groups	Participant responsiveness	Not stated				
	Mandatory Mini-CEX/DOPS	Yes	Sample	Intervention: 122 undergraduate medical trainees; Control: 183 undergraduate medical trainees	Program differentiation	Low				
	Number of Mini-CEX/DOPS	4 Mini-CEX in 8-10 weeks. Maximum of Mini-CEX allowed: 1 per week			Monitoring of control	Yes				
	Assessment sheet	7 sub-scales, one global rating, space for narrative comments regarding trainee's strengths, weaknesses and action plan			Program reach	All students in Internal Medicine clerkship *(high)*				
	Feedback provider	66 internists from main teaching hospital or affiliated hospitals. Supervisors were paid for doing the Mini-CEXs.			Adaptation	Not stated				
	Similar tools	CbD, case reflections, logbooks, weekly supervisor feedback in groups								

Suhoyo et al., 2014: Neurology (38)	Institution	Faculty of Medicine, Universitas Gadjah Mada and teaching hospitals or affiliated hospitals, Indonesia	Aim of study	To investigate the effect of Mini-CEX on trainees' clinical competence	Fidelity	Not stated	No significant differences in OSLER results were found between intervention and control group (Mann–Whitney U test, Z=-0.57, p>0.05).	Level 2b	No effect of Mini-CEX on clinical performance *(medium)*	12
	Mini-CEX/DOPS	Mini-CEX	Method	Sequential cohort study; Outcome: score in OSLER	Dosage	100% *(Dosage I: high)* resulting in 0.5 Mini-CEX/week *(Dosage II: high)*				
	Purpose of Mini-CEX/DOPS	Formative and summative	Intervention	Formative and summative Mini-CEX	Quality	Not stated				
	Feedback recipient	Students in Neurology clerkship	Control	Existing assessment program in clerkships including CbD, case reflections (more than in intervention group), logbooks, weekly supervisor feedback in groups	Participant responsiveness	Not stated				
	Mandatory Mini-CEX/DOPS	Yes	Sample	Intervention: 183 undergraduate medical trainees; Control: 186 undergraduate medical trainees;	Program differentiation	Low				
	Number of Mini-CEX/DOPS	2 Mini-CEX in 2-4 weeks. Maximum of Mini-CEX allowed: 1 per week			Monitoring of control	Yes				
	Assessment sheet	7 sub-scales, one global rating, space for narrative comments regarding trainees' strengths, weaknesses and action plan			Program reach	All students in Neurology clerkship *(high)*				
	Feedback provider	22 neurologists from main teaching hospital or affiliated hospitals. Supervisors were paid for doing the Mini-CEXs			Adaptation	Not stated				
	Similar tools	CbD, case reflections, logbooks, weekly supervisor feedback in groups								

Kirkpatrick level 2b
DOPS

Study (citation number)	Setting		Study design		Implementation status		Reported outcome: educational impact of WPBA	Kirkpatrick level	Interpretation of educational impact	MERSQI
Hengameh et al., 2015 (44)	Institution	Nursing Army College, Tehran, Iran	Aim of study	To compare the effect of DOPS on clinical skills of nursing students	Fidelity	Not stated	Intervention group had significantly higher checklist scores than control (IV catheterization: t-test, T=-10.373, p=0.000; change dressing: t-test, T=-11.858, p=0.000)	Level 2b	Positive effect of DOPS on clinical performance *(high)*	11.5
	Mini-CEX/DOPS	DOPS	Method	Randomized controlled trial; Outcome: validated and reliable assessment checklists for two procedures (Intravenous catheterization and changing dressing)	Dosage	Not stated				
	Purpose of Mini-CEX/DOPS	Formative	Intervention	Formative DOPS	Quality	Not stated				
	Feedback recipient	Nursing students in medical-surgical and intensive courses	Control	Routine method including a subjective judgment of an instructor about general skills of the student during their clinical course	Participant responsiveness	Not stated				
	Mandatory Mini-CEX/DOPS	Not stated	Sample	70 undergraduate nursing trainees	Program differentiation	High				
	Number of Mini-CEX/DOPS	3 DOPS			Monitoring of control	Yes				
	Assessment sheet	Not stated			Program reach	Not stated				
	Feedback provider	10 clinical instructors			Adaptation	Not stated				
	Similar tools	None								
Roghieh et al., 2013 (9)	Institution	Amol School of Nursing, Iran	Aim of study	To investigate the effect of DOPS on trainees' learning	Fidelity	Not stated	The intervention group showed significantly higher skill levels in arterial blood sampling and endotracheal suctioning than the control group (Arterial blood sampling: U test, U=19.07,p<0.001; entotracheal suctioning: U test, U=9.05, p<0.001).	Level 2b	Positive effect of DOPS on clinical performance *(high)*	12.5
	Mini-CEX/DOPS	DOPS	Method	Semi-experimental randomized controlled study; Outcome: skill level in arterial blood sampling and endotracheal suctioning measured with checklist (maximum of 20 points)	Dosage	Not applicable				
	Purpose of Mini-CEX/DOPS	Formative	Intervention	Formative DOPS in addition to routine logbook assessment	Quality	Not stated				
	Feedback recipient	4th-year nursing students	Control	Routine logbook assessment	Participant responsiveness	Not stated				
	Mandatory Mini-CEX/DOPS	Yes	Sample	Intervention: 20 undergraduate nursing trainees; Control: 19 undergraduate nursing trainees	Program differentiation	Medium				
	Number of Mini-CEX/DOPS	Not stated			Monitoring of control	Yes				
	Assessment sheet	6 sub-scales, one global rating, space for narrative comments regarding stregnth, area for improvement and action plan			Program reach	100% *(high)*				
	Feedback provider	Nurses			Adaptation	Not stated				
	Similar tools	Logbook								

Supplement 3: Studies included in systematic review and extracted data. Classification of extracted data is displayed in italics. Abbreviations: ACE (assessment of clinical expertise), CbD (case based discussion), CP (case conference), FY (foundation year), ITER (in-training evaluation reports), JCP (journal club presentation), Mini-ACE (mini-assessed clinical encounter), Mini-PAT (mini-peer assessment tool), MSF (multisource feedback), OSCE (objective structured clinical examination), OSLER (objective structured long examination record), PDA (personal digital assistant), PSQ (patient satisfaction questionnaire), WPBA (workplace-based assessment)
